# 862. Machine Learning to Differentiate Group A Streptococcal (GAS) from Non-GAS Pharyngitis Using a Custom Smartphone App

**DOI:** 10.1093/ofid/ofad500.907

**Published:** 2023-11-27

**Authors:** Rana F Hamdy, Trong Nguyen, Emily Ansusinha, Youness Arjoune, Jeffrey S Dome, Raj Shekhar

**Affiliations:** Childrens National Hospital, Washington, District of Columbia; Children's National Research Institute, Washington, District of Columbia; Children's National Hospital, Washington, District of Columbia; Children's National Research Institute, Washington, District of Columbia; Children’s National Hospital, Washington, District of Columbia; Children's National Hospital, Washington, District of Columbia

## Abstract

**Background:**

Sore throat is one of the top three reasons for ambulatory pediatric visits in the United States. Approximately one-third of children with sore throat are diagnosed with GAS pharyngitis, for which antibiotics are indicated. Because symptoms and physical exam-based clinical prediction rules are insufficient to accurately diagnose GAS pharyngitis, laboratory testing is needed for conclusive diagnosis. Antibiotics are frequently overprescribed for pharyngitis, especially in the telemedicine setting where testing is not readily at hand. We developed a custom mobile app, StrepApp, to capture throat images and apply machine learning (ML) to distinguish GAS pharyngitis from other causes of sore throat.

**Methods:**

We enrolled patients >3 years old presenting with sore throat in the Children’s National Emergency Department. Using StrepApp, we collected throat images and presenting clinical symptoms. We defined GAS pharyngitis as a positive microbiologic GAS test and absence of upper respiratory infection (URI) symptoms; non-GAS pharyngitis was defined as those with negative microbiologic tests. We excluded patients who had received an antibiotic active against GAS in the previous 48 hours, those whose captured images were of poor quality or those with incomplete data, missing labs, or a positive GAS test in a patient presenting with URI symptoms. For ML classification, Scale Invariant Feature Transform (SIFT) was applied to throat images. Visual features were combined with clinical symptoms as input into a support vector machine (SVM) classifier to make a GAS versus non-GAS determination (Figure 1). We calculated sensitivity, specificity, positive predictive value (PPV), and negative predictive value (NPV) of the trained classifier.

**Figure:**
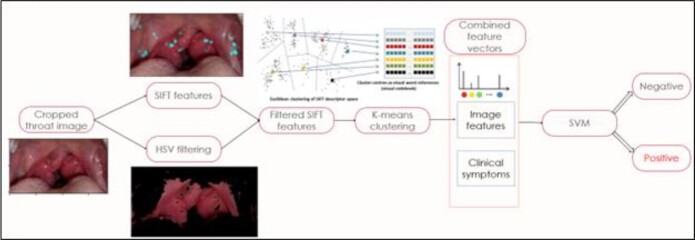

**Results:**

Of 262 patients enrolled, 148 images were analyzed. Curated images were split into 77 training images and 33 test images (Figure 2). The overall accuracy of the SVM classifier was 93.9%. The sensitivity, specificity, PPV, and NPV were 50.0%, 96.8%, 50.0%, and 96.8%, respectively.

**Figure:**
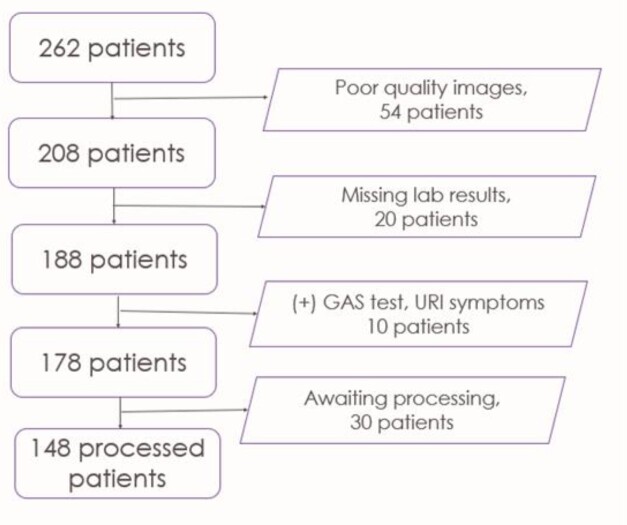

**Conclusion:**

StepApp shows promise to distinguish GAS from non-GAS pharyngitis with high specificity. The small sample size of the database allowed only conventional machine learning. As the database expands, deep learning with superior results may be possible.

**Disclosures:**

**All Authors**: No reported disclosures

